# The association between reduced hypothalamic subregion volume and aortic aneurysm risk: a Mendelian randomization and multi-omics study with clinical MRI validation

**DOI:** 10.3389/fcvm.2026.1859362

**Published:** 2026-08-06

**Authors:** Xiangfeng Huang, Buyu Ke, Bo Yin

**Affiliations:** 1 Second Affiliated Hospital and Yuying Children’s Hospital of Wenzhou Medical University, Zhejiang, China; 2 Wenzhou Medical University, Zhejiang, China

**Keywords:** aortic aneurysms, hypothalamus, inflammation, magnetic resonance imaging, multi-omics

## Abstract

**Background:**

Aortic aneurysms represent significant cardiovascular pathologies with an incompletely understood pathogenesis. The hypothalamus, a critical structure in regulating autonomic functions, endocrine activity, and circadian rhythms, may play a role in this context.

**Methods:**

We used Mendelian randomization (MR) to test the causal effects of hypothalamic subregional volumes on aortic aneurysm risk. Utilizing T1-weighted MRI data from individuals with aortic aneurysms and healthy controls, we investigated group differences in the volumes of five hypothalamic subregions. Multiple analytical methods were combined to pinpoint the causative gene, followed by mediation analysis with cis-expression quantitative trait loci targets.

**Results:**

MR analysis revealed that hypothalamic volume reduction was genetically linked to higher aortic aneurysm risk: anterior superior (OR = 0.33, 95%CI: 0.13-0.81, FDR = 0.026), inferior tubular (OR = 0.52, 95%CI: 0.33-0.83, FDR = 0.026), and superior tubular (OR = 0.36, 95%CI: 0.16-0.80, FDR = 0.026). Brain MRI showed reduced superior tubular volume in aortic aneurysm patients versus controls (173.22 ± 20.99 mm^3^ vs 185.87 ± 20.90 mm^3^; *p* < 0.05, 95%CI: 0.941-0.998). Multiple analytical approaches identified candidate genes potentially related to aortic aneurysm and suggested that IL1B may act as a potential statistical mediator in the association between hypothalamic volume and aortic aneurysm risk.

**Conclusions:**

The findings suggest that genetically predisposed structural changes in the hypothalamus, especially reduced superior tubular volume, are associated with a higher risk of aneurysms, and imply that the hypothalamus may affect aortic aneurysm development through an inflammatory pathway.

## 1 Introduction

Aortic aneurysm (AA) represents a significant cardiovascular pathology, characterized by localized dilation of the aorta and an imbalance in wall tension, which may precipitate rupture and is associated with elevated rates of mortality and morbidity (1). While hypertension, smoking, atherosclerosis, and genetic predisposition are recognized as critical risk factors (2, 3), the complete pathogenesis of aortic aneurysms remains incompletely understood. Recent research has shifted its focus towards regulatory mechanisms beyond the structural components of the vascular wall, including autonomic nervous system regulation, metabolic homeostasis, and disturbances in circadian rhythm (4–6). Given its central role in regulating the autonomic nervous system, endocrine functions, and circadian rhythms (7), the hypothalamus is posited to play a pivotal role in the pathogenesis of aortic aneurysms.

Currently, in the field of aortic aneurysm research, the morphological and volumetric changes of the hypothalamus have not been extensively investigated. Nevertheless, there is evidence suggesting that the structure and function of the hypothalamus may be associated with the homeostasis of the cardiovascular system (8, 9). The extent to which these hypothalamic alterations contribute to the formation of aortic aneurysms, as opposed to being secondary responses in the progression of the disease, remains unclear. Establishing causal relationships is crucial for determining the potential of the hypothalamus as a therapeutic or preventive target. Furthermore, most studies treat the hypothalamus as a homogeneous entity, overlooking the structural and functional heterogeneity of its various subregions, which may be critical for elucidating the specific mechanisms involved.

In addition to structural and functional factors, aortic aneurysms exhibit a significant hereditary component, with numerous genome-wide association studies (GWAS) identifying various susceptibility loci for this condition (10, 11). Notably, many single-nucleotide polymorphisms (SNPs) associated with these loci are located in non-coding regions, suggesting a potential role in regulating gene expression. The expression-regulated cis-expression quantitative trait loci (cis-eQTL) serve as a crucial link for elucidating the non-coding GWAS signaling pathway, effectively connecting genetic variation, gene expression, and phenotype, thereby providing insights into causal mechanisms (12). Nonetheless, the extent to which cis-eQTLs may mediate the influence of hypothalamic structures on the susceptibility to aortic aneurysms remains unclear.

Mendelian randomization (MR) employs genetic variation as an instrumental variable to deduce the causal relationship between an exposure and a disease outcome (13). The fundamental assumptions of MR include: the instrumental variables (IVs) are strongly associated with the exposure; they are independent of confounding factors; and their effect on the outcome is mediated exclusively through the exposure (14). By integrating GWAS data on aortic aneurysms with imaging-derived hypothalamic phenotypes, this study aims to investigate whether structural alterations in the hypothalamus play a causal role in the pathophysiology of aortic aneurysms. Concurrently, advancements in deep learning have enabled precise segmentation of brain structures in neuroimaging. A recent study utilized deep convolutional neural networks to segment the hypothalamus into five distinct subregions using MRI data, uncovering their genetic and functional heterogeneity (15). This methodological advancement provides a foundation for exploring the specific roles of hypothalamic subregions in the context of aortic aneurysms.

Building upon the aforementioned advancements, this study sought to elucidate the role of hypothalamic subregions in the susceptibility to aortic aneurysms. Initially, a two-sample MR analysis was conducted to investigate the causal relationship between volumetric changes in five distinct hypothalamic subregions and aortic aneurysms. Subsequently, brain MRI data were employed to assess the association between these structural alterations and aortic aneurysms. Following this, differential expression and functional enrichment analyses at the transcriptomic level were performed to identify candidate gene sets implicated in the development of aortic aneurysms and to explore their potential molecular pathways. These genes were further examined through subsequent gene expression and colocalization analyses to refine the identification of causal genes. Finally, the study explored whether cis-eQTLs associated with these structural changes serve as potential mediators in the hypothalamic-aortic aneurysm pathway.

## 2 Materials and methods

### 2.1 Study design

**Figure 1:**
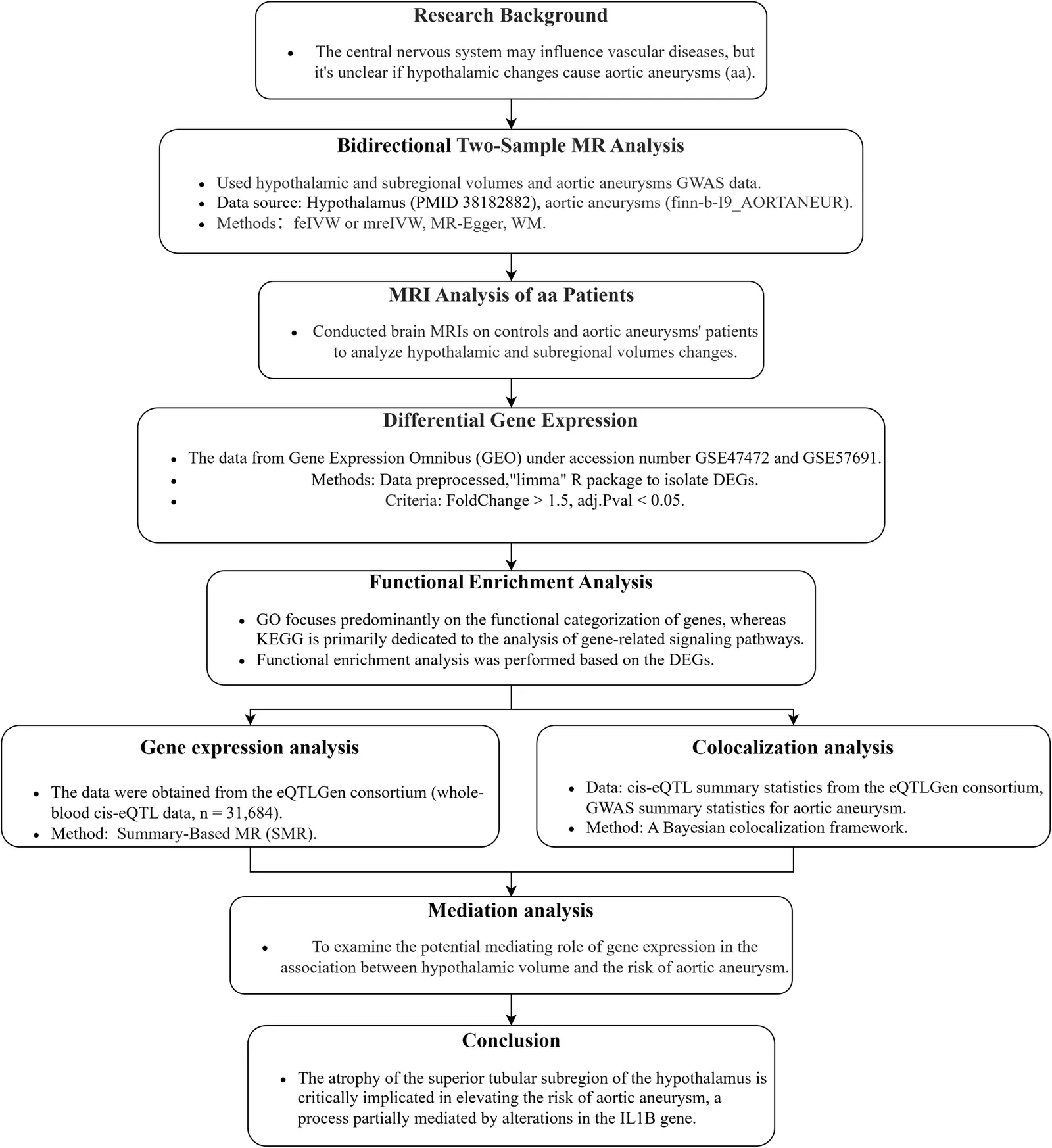
Research methodology flowchart.

Figure 1 shows the flowchart design of this study.

### 2.2 GWAS of the hypothalamus and aortic aneurysm

GWAS summary statistics pertaining to hypothalamic volume and its five subregions were obtained from a prior investigation that utilized MRI and genetic data from a cohort of 32,956 individuals (16). This prior study, which employed the same segmentation methodology as the current research and conducted GWAS, identified 23 loci associated with hypothalamic volume. These loci are enriched with genes implicated in intracellular trafficking and steroid metabolism.

GWAS summary statistics for aortic aneurysms were obtained from the GWAS summary statistical dataset finn-b-I9_AORTANEUR, as published by the FinnGen project, version R9 (N_case = 2,825; N_control = 206,541). FinnGen is a large-scale genomic study of the Finnish population that combines genetic data from approximately 500,000 individuals with health information from the National Health Registry. Aortic aneurysm cases were identified using ICD-10 code I71, while controls lacked this condition. The study was ethically approved, and informed consent was obtained from all participants.

### 2.3 Mendelian randomization analysis

MR analysis was conducted using the GWAS summary statistics described in Section 2.2, including hypothalamic volume GWAS data as the exposure and aortic aneurysm GWAS data as the outcome. We used the TwoSampleMR package to evaluate bidirectional causal relationships between the volumes of hypothalamic regions and the risk of aortic aneurysm. In the forward MR analysis, hypothalamic volume was the exposure and aortic aneurysm the outcome, while the reverse analysis swapped these roles. Genetic IVs were defined as genome-wide significant (*p* < 5 × 10−8) SNPs with a minor allele frequency (MAF) > 0.01. To reduce bias from weak instruments, only SNPs with F-statistics > 10 were retained (17). The resulting genetic IVs were pruned to high independence with an r2 threshold of 0.001 and a window size of 10 Mb. For both the exposure and outcome GWAS datasets, SNPs lacking essential information, such as effect sizes, standard errors, or allele details, were excluded before harmonization. Ambiguous or palindromic variants were corrected or excluded during harmonization to ensure allele alignment.

Causal estimates were obtained using the inverse variance-weighted (IVW) method, with a random-effects model for traits with more than three instruments and a fixed-effects model for those with two or three (18). Where only one SNP was available, the Wald ratio was applied (19). Odds ratios (ORs) for disease risk were reported per standard deviation (SD) increase in hypothalamic volume. The false discovery rate (FDR) based on the Benjamini-Hochberg approach was used for multiple testing corrections. A significance level of FDR-corrected *p* < 0.05 was considered significant.

Sensitivity analyses were performed to assess potential pleiotropy and heterogeneity. Horizontal pleiotropy was evaluated using MR-Egger regression and the MR-PRESSO Global test (20, 21), while Cochran's Q statistic was used to assess heterogeneity among instrumental variables (22). In addition, Steiger filtering was applied to verify the causal direction, ensuring that the genetic variants explained more variance in the exposure than in the outcome.

Supplement 2 contains a complete STROBE-MR checklist to ensure thorough and transparent reporting of Mendelian randomization studies.

### 2.4 Validation of hypothalamic structural calculations in cohorts

The study included 30 patients with aortic aneurysms diagnosed via CT or MR imaging at the Second Affiliated Hospital of Wenzhou Medical University from January 2022 to August 2025. Abdominal aortic aneurysms (AAA) were defined as an aorta diameter ≥ 3.0 cm, and thoracic aortic aneurysms (TAA) as a segment exceeding the reference limit or ≥ 4.0 cm. Patients were classified using ICD-10 codes: I71.200 for unruptured thoracic and I71.400 for unruptured abdominal aneurysms. A healthy control group of 30 individuals was selected from 200 screened at our imaging center, excluding those with neurological or psychiatric disorders, cranial trauma or surgery, and poor MRI quality. Using a 1:1 gender and age (± 2 years) matching with the patient group, we selected 30 HCs for the study.

Hypothalamic volumes and those of its five subregions (anterior superior, anterior inferior, superior tuberal, inferior tuberal, and posterior) were determined using an automated segmentation tool (15). The tool employs a 3D U-Net architecture, trained on T1-weighted MRI scans from 37 manually labeled subjects. To augment the training dataset, data augmentation was applied to the MRI scans. The U-Net model integrates both low-level spatial details and high-level semantic information via its symmetric encoder–decoder design with skip connections, enabling accurate segmentation of complex anatomical structures. The method demonstrated high reproducibility without additional preprocessing. Total hypothalamic volume was calculated as the sum of the subregional volumes.

Importantly, the MRI-based analysis was prespecified as an independent validation step based on the Mendelian randomization results. Accordingly, statistical comparisons were restricted to the hypothalamic subregions that showed evidence of a causal association in the MR analysis, thereby reducing the multiple-testing burden and ensuring hypothesis-driven validation.

### 2.5 MRI scan

A 3.0 Tesla magnetic resonance imaging scanner (Ingenia Elition, Philips Medical, Best, the Netherlands) was used to acquire the MRI images. 3D T1-weighted images was acquired using a 3D turbo-fast echo sequence. The parameters were as follows: TR/TE = 8.1/ 3.7 ms; slices = 170; thickness = 1 mm; gap 0 mm; FA = 8◦; acquisition matrix = 256 × 256; FOV = 256 mm × 256 mm.

### 2.6 Statistical analysis for demographic, clinical, and neuroimaging data

Statistical analysis was performed using IBM SPSS Statistics 27.0. The Shapiro–Wilk test was used to assess the normality of continuous variables. Normally distributed data are shown as mean ± SD, while non-normally distributed data are presented as median and IQR. Categorical variables are given as frequencies. In the analysis of baseline data, a paired sample t-test was employed for paired continuous variables that adhered to a normal distribution. For paired continuous variables that deviated from normal distribution assumptions, the Wilcoxon signed-rank test was utilized. The McNemar test was applied to assess paired categorical variables. In the comparison of three groups (healthy controls HC, abdominal aortic aneurysm AAA, thoracic aortic aneurysm TAA), One-way ANOVA was applied for normally distributed variables with homogeneous variance. If these conditions weren't met, the Kruskal–Wallis H test was used for non-parametric analysis.

A linear regression model was employed to compare hypothalamic subregion volumes between the healthy control (HC) and aortic aneurysm groups. The analysis was restricted to candidate subregions identified by Mendelian randomization, with adjustments for age, sex, body mass index (BMI), hypertension, diabetes, and total intracranial volume (TIV).

### 2.7 Differential gene expression

Gene expression datasets related to aortic aneurysm were retrieved from the Gene Expression Omnibus (GEO) database. GSE47472 contains human abdominal aortic aneurysm neck tissue samples from 14 AAA patients and 8 non-aneurysmal aortic control samples from organ donors. GSE57691 contains human abdominal aortic tissue samples from 49 AAA patients, including 20 small AAA and 29 large AAA samples, and 10 non-aneurysmal control aortic samples from organ donors (23, 24). Differential expression analysis was performed by comparing AAA aortic tissue samples with non-aneurysmal control aortic tissue samples. For GSE57691, both small and large AAA samples were combined into a single AAA group. Notably, these transcriptomic datasets were derived from AAA tissues. Although the primary outcome of this study includes both AAA and TAA, AAA has a higher incidence (25) and shares key pathological mechanisms with TAA, particularly inflammation and extracellular matrix remodeling (26). Therefore, AAA-based transcriptomic profiles were used to capture core molecular features of aortic aneurysm.

All datasets were obtained from the GEO database, with microarray data downloaded in the MINiML format, which contains complete information on platforms, samples, and records within each GSE. For datasets that were not normalized, log₂ transformation was first applied. If standardization had not been performed, quantile normalization was conducted using the normalize. Quantiles function in the preprocess core package of R. Probe IDs were mapped to gene symbols based on platform annotation files; probes corresponding to multiple genes were excluded, and the average expression value was calculated for genes represented by multiple probes. For datasets generated on the same platform but across different batches, batch effects were corrected using the removeBatchEffect function in the limma package of R.

For the integrated analysis of different datasets or datasets generated from multiple platforms, common gene symbols across datasets were first extracted. Different datasets or platforms were then labeled as distinct batches, and batch effects were removed using the removeBatchEffect function in the limma package. The effectiveness of data normalization was evaluated using boxplots, while the removal of batch effects was assessed by comparing principal component analysis (PCA) plots before and after batch correction. Eventually, differential expression analysis was performed using the limma package. Differentially expressed genes (DEGs) were identified using the criteria of fold change > 1.5 and adjusted *P* < 0.05.

### 2.8 Functional enrichment analysis

Functional enrichment analysis was performed based on the DEGs identified in Section 2.7. Gene Ontology (GO) enrichment analysis was conducted to annotate gene functions across the Biological Process, Molecular Function, and Cellular Component categories. Kyoto Encyclopedia of Genes and Genomes (KEGG) pathway enrichment analysis was performed to explore biological pathways associated with these DEGs.

The clusterProfiler package in R was used for GO and KEGG enrichment analyses. Statistical analysis was conducted using R software, version 4.0.3. Enrichment results with a p-value < 0.05 were considered statistically significant.

### 2.9 Gene expression analysis

In our study, gene expression data were obtained from the eQTLGen consortium, which includes 31,684 peripheral whole blood samples from individuals of predominantly European ancestry across multiple population-based cohorts (27). The gene expression measurements were derived from bulk RNA sequencing of whole blood, rather than isolated blood cell subtypes. It should be noted that the eQTLGen dataset does not contain phenotypic data on aortic aneurysm or hypothalamic volume. Instead, it provides summary-level associations between genetic variants and gene expression levels (cis-eQTLs). The summary-based Mendelian randomization (SMR) method was used to integrate cis-eQTL data from the eQTLGen consortium with GWAS summary statistics for aortic aneurysm. This approach allows the identification of genes whose expression levels are associated with these traits through shared genetic variants (28, 29). To account for multiple testing across all tested probes, FDR correction was applied. Given the exploratory nature of whole transcriptome screening in this study, results based on nominal SMR p-values (*P* < 0.05) were also reported to prioritize candidate genes for subsequent colocalization analysis. The HEIDI test was used to assess heterogeneity; *P* > 0.05 indicates no evidence of linkage or pleiotropy.

### 2.10. Colocalization analysis

The colocalization analysis was performed to assess whether the genetic signals regulating gene expression and those associated with aortic aneurysm share a common causal variant. Specifically, cis-eQTL summary statistics from the eQTLGen consortium (as described in Section 2.9) were integrated with GWAS summary statistics for aortic aneurysm (Section 2.2). A Bayesian colocalization framework was applied to evaluate five hypotheses: H0 (no association), H1 (association with the first trait), H2 (association with the second trait), H3 (distinct causal variants for each trait), and H4 (a shared causal variant for both traits) (30, 31).

The prior probabilities were set to standard coloc values: p1 = 1e-4, p2 = 1e-4, and p12 = 1e-5. To assess the robustness of colocalization evidence to prior assumptions, we conducted a sensitivity analysis over a range of p12 values from 1e-5 to 1e-3, while keeping p1 and p2 fixed at 1e-4. Posterior probabilities for all five hypotheses were reported. In addition to PPH4, we calculated the conditional colocalization probability, defined as H4/(H3 + H4), which represents the probability of a shared causal variant conditional on evidence supporting association with both traits in the region (32, 33). A conditional probability H4/(H3 + H4) ≥ 0.7 was considered supportive evidence for colocalization. A gene was considered to have evidence of colocalization with disease only if the evidence remained consistent across all tested p12 values, defined as either PPH4 > 0.80 or H4/(H3 + H4) ≥ 0.70 at each p12 setting.

### 2.11. Mediation analysis

We conducted a two-step MR-mediated analysis to explore whether significant genes influence the link between hypothalamic volume and the risk of aortic aneurysm. We utilized eQTL data from the eQTLGen consortium (27), focusing on cis-eQTLs, which are genetic variations within 1 Mb of the gene's transcription start site. IVs were selected based on these criteria: *p*-value < 5e-8, ClumpingWindow: 10,000 kb, r2 < 0.001. Initially, we evaluated the causal effect of hypothalamic volume on key genes using MR, employing a genome-wide significance threshold (*p* < 5e-6) to identify IVs for the volume of the Superior tubular. Subsequently, we examined the causality of key genes on aortic aneurysms, applying FDR correction for multiple testing.

Mediation was assessed utilizing the “product of coefficients” approach, wherein indirect effects were determined by calculating the product of Mendelian randomization estimates from hypothalamic volume to key genes and from these key genes to aortic aneurysms. Standard errors were estimated using the delta method (34), and the proportion mediated was defined as the ratio of the indirect effect to the total effect. A *p*-value of less than 0.05 was considered indicative of statistical significance in mediation analysis.

## 3 Results

### 3.1 Bidirectional MR analysis and sensitivity analysis

A forward MR analysis indicates a significant causal relationship between reduced volume in three hypothalamic subregions—Anterior superior (OR = 0.33, 95%CI: 0.13-0.81, FDR = 0.026), Inferior tubular (OR = 0.52, 95%CI: 0.33-0.83, FDR = 0.026), and Superior tubular (OR = 0.36, 95%CI: 0.16-0.80, FDR = 0.026)—and an increased risk of aortic aneurysm (Supplementary Figure S1A, Supplementary Table S1). Conversely, a reverse MR analysis demonstrates that aortic aneurysms do not exhibit a causal association with volumetric changes in the hypothalamus or its five subregions (Supplementary Figure S1B, Supplementary Table S2). See Supplementary Table S10 for detailed instrument variable information.

In the sensitivity analysis, Cochran's Q test revealed partial heterogeneity in the anterior superior-aortic aneurysm analysis (Q = 7.676, *P* = 0.006), indicating variability in effect sizes across the instrumental variables, whereas no significant heterogeneity was observed in the other analyses. Furthermore, both the MR-Egger intercept (*p* > 0.05) and the MR-PRESSO global test (*p* > 0.05) suggested the absence of horizontal pleiotropy (Supplementary Table S3). In addition, Steiger filtering indicated a consistent causal direction for all instrumental variables (Supplementary Table S10).

Based on these findings, and considering the presence of heterogeneity in the anterior superior region but not in the inferior tubular and superior tubular regions, subsequent analyses were focused on these two hypothalamic subregions.

### 3.2 Clinical cohort validation of Mendelian randomization results

**Figure 2:**
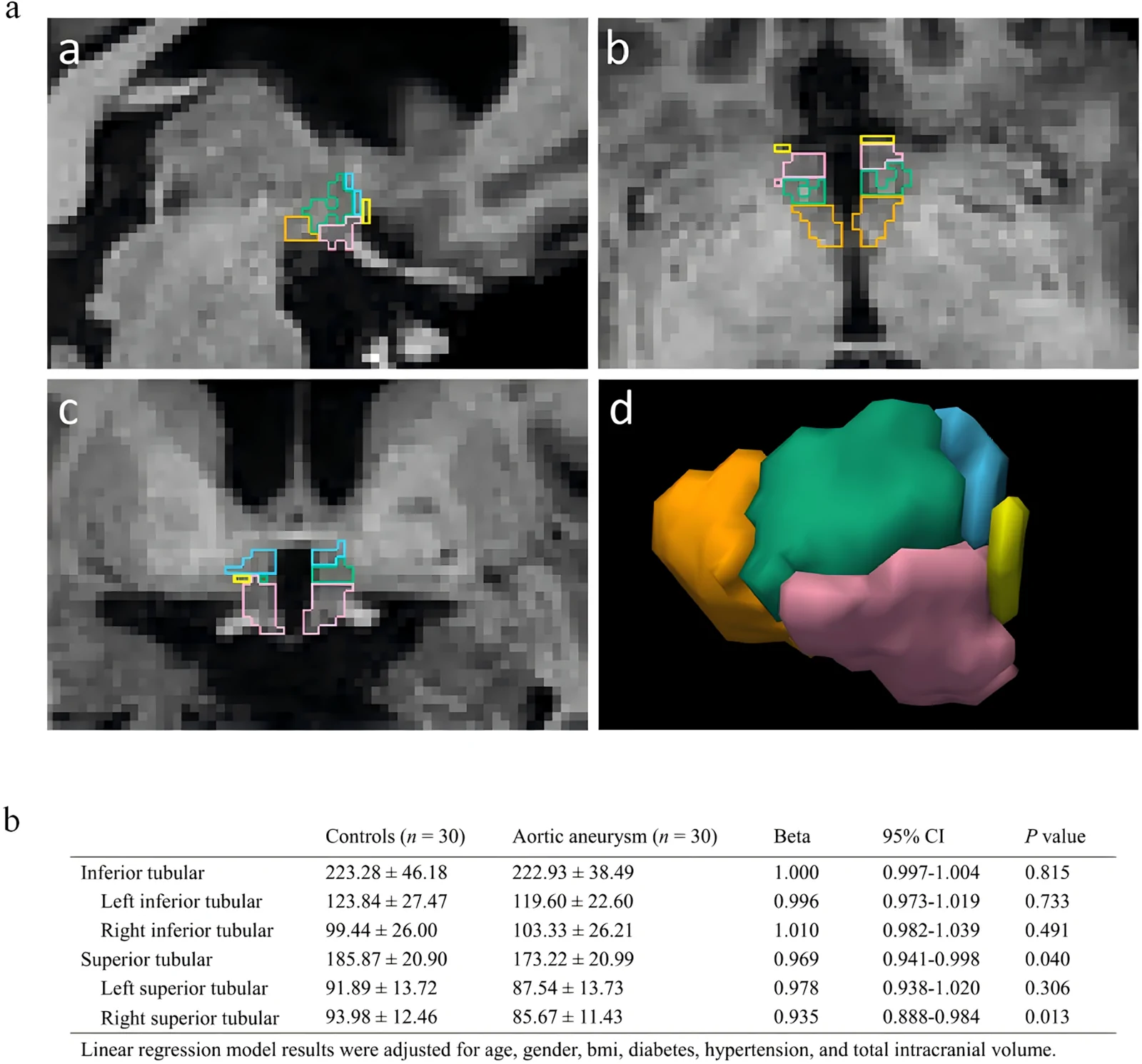
A reprinted from Billot B et al. (15), under CC BY 4.0 license. a shows a manually segmented hypothalamus in **(a)** sagittal, **(b)** axial, and **(c)** coronal views, with **(d)** a 3D rendering of the right hypothalamus. Subunits are color-coded: anterior superior (blue), anterior inferior (yellow), superior tubular (green), inferior tubular (pink), and posterior (orange). b compares the volume differences of each hypothalamus subregion between the AA and HC groups. Figure 2

Figure 2 a shows the spatial features of hypothalamic subregion segmentation. Supplementary Table S11 lists the nuclei in each hypothalamic subregion. To validate previous MR findings, we used MRI data from 30 patients with aortic aneurysms (11 with AAA, 19 with TAA) and 30 healthy controls (HCs). We combined AAA and TAA patients into a single aortic aneurysm group and compared them with the HC group for differences in hypothalamic volume. Supplementary Table S10 details the participants' demographic and clinical characteristics. Statistical analysis found no significant differences in age, sex, BMI, diabetes, hypertension, or total intracranial volume between the two groups (*p* > 0.05). The AA group had inferior tubular region volumes identical to those of the control group (222.93 vs. 223.28 mm^3^, *P* = 0.815, 95%CI: 0.997-1.004). However, the superior tubular region volume was considerably lower in the AA group than in the control group (173.22 vs. 185.87 mm^3^, *P* = 0.040, 95%CI: 0.941-0.998) (Figure 2b). This suggests a significant reduction in the volume of the superior tubular region in AA patients, consistent with Mendelian randomization findings.

Exploratory subgroup analyses were performed to assess potential heterogeneity across aneurysm subtypes (AAA vs. TAA) and hypothalamic hemispheric laterality. These analyses were not part of the prespecified primary hypothesis and are therefore presented as descriptive and hypothesis-generating. When comparing hypothalamic whole and subregion volumes across the three groups, significant differences were observed in the superior tubular subregion (F = 6.956, *P* = 0.002). Further side-specific analyses revealed significant group differences in the right superior tubular region (F = 7.373, *P* = 0.001) (Supplementary Table S12). In the comparative analysis of regional indicators, significant differences were found in the superior tubular region (*P* = 0.002), with the AAA group having lower values than both the HC (*P* = 0.001) and TAA groups (*P* = 0.006). The right superior tubular region exhibited particularly pronounced differences (*P* = 0.001), with the AAA group also significantly lower than the HC (*P* = 0.001) and TAA groups (*P* = 0.007). Apart from that, the left superior tubular region showed significance between the HC and AAA groups (*P* = 0.050).

### 3.3 Identification of candidate genes and results of enrichment analysis

Differential expression analysis revealed a total of 329 DEGs, comprising 111 up-regulated and 218 down-regulated genes (Supplementary Figure S2, Supplementary Table S4).

GO and KEGG enrichment analyses of differentially expressed genes in aortic aneurysm tissue revealed the following: Up-regulated genes were enriched in GO biological processes, including regulation of the acute inflammatory response, inflammatory response, response to bacterial lipopolysaccharide, and migration of neutrophils and macrophages. Molecular function terms included oxidoreductase activity and peroxidase activity, and cellular component terms were enriched in secretory granule membranes and related structures. KEGG pathway analysis showed significant enrichment of immune- and inflammation-related pathways, including the IL-17 signaling pathway, TNF signaling pathway, Toll-like receptor signaling pathway, JAK-STAT signaling pathway, and cytokine–cytokine receptor interaction. Down-regulated genes were mainly enriched in GO terms related to ribosome assembly, mitochondrial electron transport, RNA splicing, and RNA metabolism, with molecular functions involving structural ribosomal proteins and RNA binding, and cellular components localized to the ribosome and mitochondria. KEGG pathways for these genes were enriched in oxidative phosphorylation, the ribosome, the spliceosome, and several neurodegenerative disease pathways associated with mitochondrial dysfunction.

Overall, GO and KEGG enrichment analyses indicate that differentially expressed genes in aortic aneurysm tissue exhibit a global up-regulation of immune and inflammatory pathways, accompanied by a down-regulation of metabolic and protein synthesis pathways (Supplementary Figure S2, Supplementary Tables S4, S5).

### 3.4 Results of gene expression analysis

The SMR analysis identified several genes whose expression levels showed nominal associations with aortic aneurysm at P_SMR_ < 0.05, including *ADAMTS8*, *CCN3*, *COX6C*, *GSDMB*, *HSBP1*, *IL1B*, *JAG1*, *KDELR1*, *PPP1R14A*, *RSL1D1*, *SEMA4A*, *SFI1*, *SPIB*, and *TMED6*. After false discovery rate (FDR) correction for multiple testing across all tested probes, ADAMTS8 and CCN3 remained statistically significant, whereas other genes, including IL1B, did not (Supplementary Table S6). Given the multiple-testing burden and the exploratory nature of SMR in this study, these findings should be interpreted as hypothesis-generating signals. The identified genes provide candidates for further investigation using colocalization.

### 3.5 Results of colocalization analysis

Across the tested prior settings, four genes—ADAMTS8, CCN3, GSDMB, and IL1B—consistently showed colocalization with aortic aneurysm. Specifically, these genes satisfied the predefined criteria across all p12 values, showing either strong posterior support for a shared causal variant (PPH4 > 0.80) or high conditional probability favoring a shared over a distinct causal variant [H4/(H3 + H4) ≥ 0.70] (Supplementary Table S7).

Overall, these findings indicate that ADAMTS8, CCN3, GSDMB, and IL1B show robust and prior-consistent colocalization signals with aortic aneurysm, supporting their potential roles as candidate genes underlying shared genetic architecture.

### 3.6 Mediation effect of hypothalamic volume on aortic aneurysm via cis-eQTL

**Figure 3:**
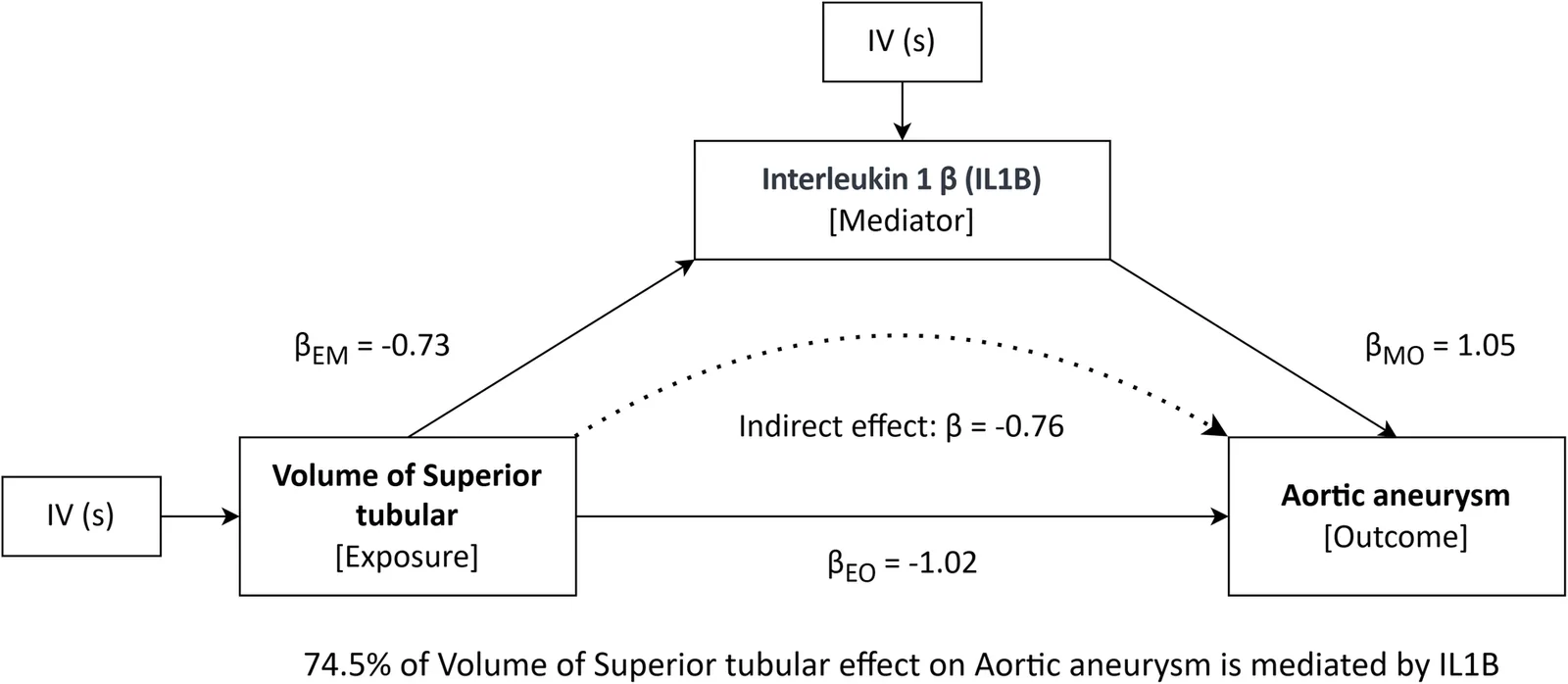
IL1B mediates the link between superior tubular volume and aortic aneurysm. Abbreviations: β_EM_, effects of exposure on mediator; β_MO,_ effects of mediator on outcome; β_EO_, effects of exposure on outcome.

We identified associations between hypothalamic volume and IL1B expression. Specifically, decreased superior tubular volume was associated with increased IL1B expression (FDR = 0.004). The remaining results are presented in Supplementary Table S8. Subsequent analyses suggested that higher IL1B expression may be associated with an increased risk of aortic aneurysm (FDR = 0.030) (Supplementary Table S9). Mediation analysis further indicated that IL1B may act as a potential statistical mediator in the association between superior tubular volume and aortic aneurysm, accounting for a substantial proportion of the total effect (74.5%); however, this finding should be interpreted with caution given the modest statistical significance (*P* = 0.048, 95%CI: −1.514 to −0.006) (Figure 3; Supplementary Table S13).

## 4 Discussion

Despite its small size, the hypothalamus plays a crucial role in regulating essential body functions. It communicates with other brain areas to manage metabolic processes and control the autonomic nervous system, affecting circadian rhythm, appetite, blood pressure, and heart rate (35).

The superior tubular region demonstrates notable volume reduction in cases of aortic aneurysm. This area encompasses the dorsomedial nucleus (DMH), paraventricular nucleus (PVN), and lateral hypothalamus (LH), all of which are integral to the regulation of autonomic, endocrine, and inflammatory processes. The DMH serves as a central hub for modulating sympathetic nerve activity. Activation of the DMH can augment the sympathetic nerve baroreflex, elevate basal sympathetic nerve activity, and subsequently increase blood pressure and heart rate, thereby enhancing vascular wall stress (36, 37).

The PVN serves as the central regulatory hub of the hypothalamic-pituitary-adrenal (HPA) axis and the sympathetic nervous system, playing a crucial role in modulating glucocorticoid secretion and suppressing inflammation via corticotropin-releasing hormone and arginine vasopressin signaling pathways (38, 39). Reduced PVN volume may disrupt this negative feedback mechanism, leading to a persistent upregulation of pro-inflammatory factors. The LH is intimately associated with energy homeostasis and the regulation of autonomic nervous functions. Orexin neurons, a fundamental neuronal type within this region, are implicated not only in energy balance (40) but also in the direct excitation of central sympathetic nerves, thereby contributing to a marked increase in blood pressure (41, 42). It is hypothesized that superior tubular volume reduction may expedite the onset and progression of aortic aneurysms via the intricate mechanism of “DMH-mediated sympathetic excitation-PVN imbalance leading to inflammation amplification and LH-induced metabolic inflammation disorder.” Additionally, the specific alterations observed on the right side imply that these mechanisms may exhibit functional lateralization.

This study demonstrated that interleukin-1 beta (IL1B) partially mediates the relationship between hypothalamic volume reduction and the risk of aortic aneurysm. Under inflammatory conditions, the hypothalamus not only expresses IL1B but also exhibits significant upregulation of IL-1 receptors, positioning the hypothalamus as a central target for inflammatory signaling (43). The regulation of IL1B in the context of aortic aneurysms can be elucidated through several mechanisms. Primarily, the involvement of IL1B in the formation of aortic aneurysms is attributable to its modulation of matrix metalloproteinases (MMPs). Research indicates that the local upregulation of IL1B correlates with increased expression of MMP-2 and MMP-9, enzymes prominently associated with anatomical aortic aneurysms and compromised biomechanical properties (44). IL1B is instrumental in the pathogenesis of TAA by altering the expression of MMP-2 and MMP-9, leading to the degradation of the aortic wall matrix, rupture of elastic fibers, and modification of the stress or strain experienced by the aortic wall (45). Moreover, the inhibition of IL1B has been shown to significantly mitigate the formation of AAA induced by angiotensin II, indicating that IL1B inhibition may offer an additional protective approach for the prevention of aortic aneurysms (46). Additionally, IL1B contributes to the progression of aneurysms by promoting apoptosis in smooth muscle cells. Empirical evidence from AAA models suggests that the upregulation of IL1B is closely linked to increased apoptosis of smooth muscle cells, potentially leading to reduced elasticity and structural degradation of the arterial wall (47). Furthermore, IL1B enhances AAA formation by inducing neutrophil extracellular trap formation (48). These findings suggest that IL1B may be a biologically plausible candidate involved in the cerebral–aortic aneurysm axis. However, the current evidence is primarily statistical and exploratory, and further studies are required to clarify its causal role and potential therapeutic relevance.

This study is subject to several limitations that warrant attention in future research. Firstly, the Mendelian Randomization analysis relies on publicly accessible databases and genetic instrumental variables. While this approach can mitigate confounding factors to some extent, the effectiveness and specificity of the instrumental variables may be limited, and the potential impact of horizontal pleiotropy on the results cannot be entirely ruled out. Secondly, the sample size for the MRI data is relatively limited, and variations in imaging protocols and data quality across different cohorts may affect the precision and comparability of hypothalamic subregion volume measurements. Third, the transcriptomic datasets used in this study were derived exclusively from AAA tissues, whereas the primary outcome was defined using a composite aortic aneurysm phenotype that may include both AAA and TAA. Although AAA is more prevalent and shares key pathological mechanisms with TAA, potential subtype-specific molecular differences may not be fully captured. In addition, the use of a composite outcome limits the ability to distinguish subtype-specific associations. Therefore, the findings should be interpreted with caution, as they may reflect shared biological pathways across aortic diseases rather than subtype-specific effects. Future studies incorporating subtype-specific transcriptomic and genetic data are warranted to further clarify these relationships. Fourth, the gene expression analysis was performed using whole-blood cis-eQTL data. As this dataset lacks phenotypic information on hypothalamic volume or aortic aneurysm, the identified gene expression associations reflect systemic genetic regulatory effects rather than tissue-specific or disease-specific mechanisms. Importantly, although FDR correction was applied in the SMR analysis to account for multiple testing, nominal P_SMR_ were used to prioritize candidate genes for subsequent colocalization analysis. This approach may introduce selection bias and increase the risk of false-positive signals being carried forward into downstream analyses. Therefore, the SMR results should be interpreted as exploratory and hypothesis-generating rather than confirmatory evidence of gene-level causality. Future studies using independent datasets and stricter multi-stage correction strategies are needed to validate these findings. Although IL1B was identified as a potential mediator in the present analysis, this finding should be interpreted with caution. The mediation effect was modest, and the analysis was based on blood-derived eQTL data rather than on hypothalamic or aortic tissue-specific expression. Therefore, IL1B should be considered as a potential statistical mediator rather than an established biological mechanism. Lastly, the study primarily focuses on a specific population, necessitating further validation of the generalizability and applicability of the results across diverse ethnic and clinical populations.

## 5 Conclusions

This study suggests that genetically predisposed alterations in the hypothalamus, particularly reduced superior tubular volume, are associated with aortic aneurysm risk and may be linked to inflammatory pathways. IL1B may represent a biologically plausible candidate involved in this association; however, its mediating role should be interpreted as exploratory and requires further validation. These findings provide preliminary insights into potential neuroimmune mechanisms underlying aortic aneurysm pathophysiology.

## Data Availability

The original contributions presented in the study are included in the article/Supplementary Material, further inquiries can be directed to the corresponding author.
