# Supplementary material for: The association between reduced hypothalamic subregion volume and aortic aneurysm risk: a Mendelian randomization and multi-omics study with clinical MRI validation
Source: Front Cardiovasc Med. 2026 Aug 6;13:1859362. doi: 10.3389/fcvm.2026.1859362 (PMC13489904; doi:10.3389/fcvm.2026.1859362)
Supplement: Supplementary file 1 [file Datasheet1.pdf]

Fig. S1

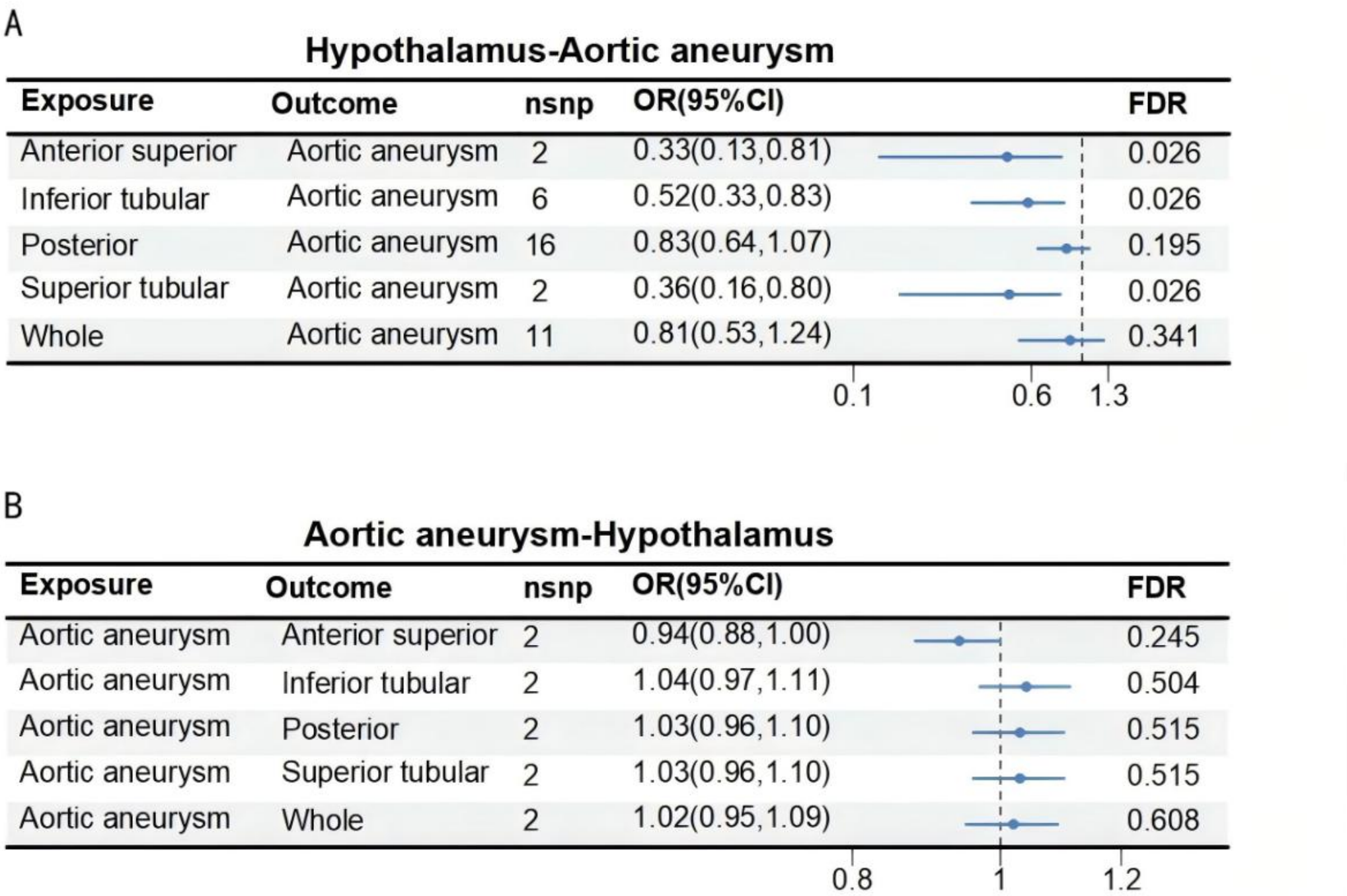

Fig. S1 presents the bidirectional MR results concerning the relationship between hypothalamic volume and aortic aneurysm. A illustrates the scenario where the volumes of each subregion of the hypothalamus were considered as the exposure, with aortic aneurysm serving as the positive MR outcome. Conversely, B depicts the situation where the aortic aneurysm was the exposure, and the volume of each hypothalamic subregion constituted the reverse MR outcome.

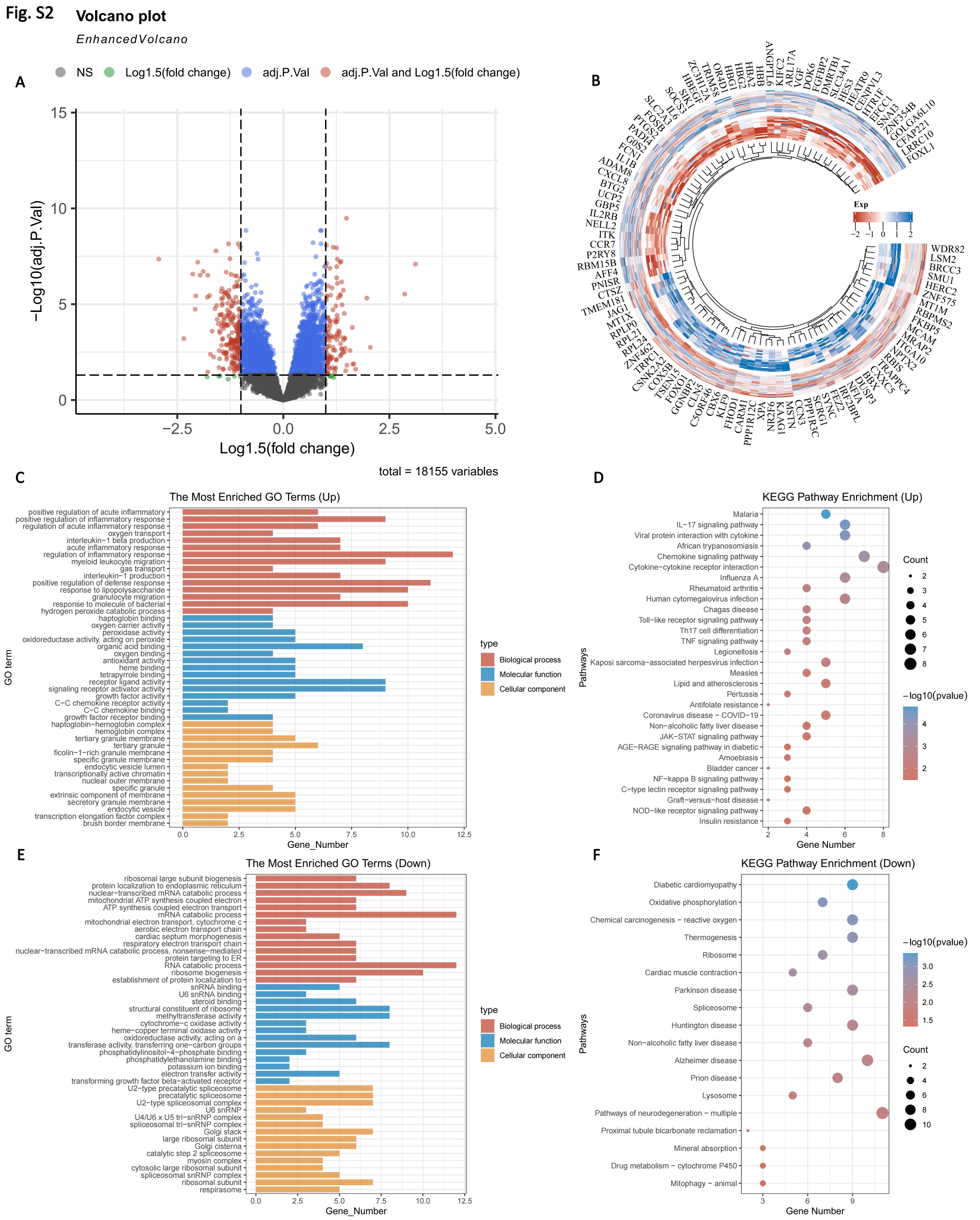

Fig. S2 A and B display volcano and heat maps of differentially expressed genes, highlighting the top 50 up- and down-regulated genes. C and D present Cnet plots for GO/KEGG enrichment of up-regulated genes, while E and F show Cnet plots for down-regulated genes.

**Table S10 Demographic and clinical data**			
	**Controls (*n* = 30)**	**AA (*n* = 30)**	***P* value**
Age (years)	68.8 ± 9.93	68.63 ± 10.18	0.949[a]
Gender (female/male)	11/19	11/19	1.000[b]
BMI	24.5 ± 3.62	23.66 ± 3.52	0.366[c]
Diabetes (Yes/NO)	8/22	5/25	0.531[b]
Hypertension (Yes/NO)	17/13	20/10	0.595[b]
Intracranial volume	1264.08 ± 145.5	1218.18 ± 103.98	0.165[c]

AAA Abdominal aortic aneurysm, *TAA* Thoracic aortic aneurysm, *BMI* Body mass index, *n* number
[a]Kruskal-Wallis test,
[b]Chi-square test,
[c]One-way ANOVA,
[d]Mann-Whitney U test,
[e]Independent samples t test

Table S11 Subregions of hypothalamic nuclei	
**Subregions**	**Nuclei included**
Anterior-superior	preoptic area; paraventricular nucleus (PVN)
Anterior-inferior	suprachiasmatic nucleus; supraoptic nucleus (SON)
Superior tubular	dorsomedial nucleus; PVN; lateral hypothalamus
Inferior tubular	infundibular (or arcuate) nucleus; ventromedial nucleus; SON; lateral tubular nucleus; tuberomamillary nucleus (TMN)
Posterior	mamillary body (including medial and lateral mamillary nuclei); lateral hypothalamus; TMN

**Table S12 Volume comparison of the hypothalamus and its subregions in aortic aneurysm subtypes**											
	**HC (*n* = 30)**	**AAA (*n* = 11)**	**TAA (*n* = 19)**	***F***	***P***	β_1	P_1	β_2	P_2	β_3	P_3
Inferior tubular	223.28 ± 46.18	218.49 ± 50.78	225.5 ± 30.58	0.094	0.911	-4.79	0.776	2.22	0.854	7.01	0.639
Left inferior tubular	123.84 ± 27.47	117.2 ± 27.63	120.99 ± 19.83	0.287	0.751	-6.63	0.498	-2.84	0.698	3.79	0.666
Right inferior tubular	99.44 ± 26	101.29 ± 33.32	104.51 ± 22.04	0.216	0.807	1.850	0.853	5.07	0.485	3.22	0.752
Superior tubular	185.87 ± 20.9	159.94 ± 22.03	180.9 ± 16.44	6.956	0.002	-25.9	0.001	-4.97	0.385	21.0	0.006
Left superior tubular	91.89 ± 13.72	81.39 ± 17.29	91.11 ± 10.06	2.610	0.082	-10.5	0.050	-0.780	0.832	9.72	0.060
Right superior tubular	93.98 ± 12.46	78.55 ± 9.52	89.8 ± 10.54	7.373	0.001	-15.4	0.001	-4.19	0.231	11.2	0.007

AAA **Abdominal aortic aneurysm,** ***TAA*** **Thoracic aortic aneurysm,** ***F*****,** ***P*** **Differences between three groups (ANCOVA).** $\beta 1$**,** $P1$**: HC vs. AAA.** $\beta 2$**,** $P2$**: HC vs. TAA.** $\beta 3$**,** $P3$**: AAA vs. TAA**

Table S13 The mediation effect of the volume of hypothalamus on aortic aneurysm via affecting cis-eQTL											
**Exposure**	**Mediator**	**Outcome**	**β_1**	**se_1**	**β_2**	**se_2**	**total_effect**	**Mediation_effect**	**se**	**p**	**Proportion**
Volume of Superior tubular	IL1B	AA	-0.73	0.22	1.05	0.43	-1.02	-0.76	0.385	0.048	74.5%

STROBE-MR checklist of recommended items to address in reports of Mendelian randomization studies[1 2]

Item No.	Section	Checklist item	Section	Relevant text from manuscript
1	**TITLE and ABSTRACT**	Indicate Mendelian randomization (MR) as the study's design in the title and/or the abstract if that is a main purpose of the study	Abstract	The abstract contains a description of Mendelian randomization (MR).
	**INTRODUCTION**			
2	**Background**	Explain the scientific background and rationale for the reported study. What is the exposure? Is a potential causal relationship between exposure and outcome plausible? Justify why MR is a helpful method to address the study question	Section 1	The central nervous system may influence vascular diseases, but it's unclear if hypothalamic changes cause aortic aneurysms.
3	**Objectives**	State specific objectives clearly, including pre-specified causal hypotheses (if any). State that MR is a method that, under specific assumptions, intends to estimate causal effects	Fig. 1	This study used two-sample MR to explore the causal link between hypothalamic subregions and aortic aneurysms, identifying intermediate genes that might mediate this influence through a two-stage mediated MR framework.
	**METHODS**			
4	**Study design and data sources**	Present key elements of the study design early in the article. Consider including a table listing sources of data for all phases of the study. For each data source contributing to the analysis, describe the following:		
	a)	Setting: Describe the study design and the underlying population, if possible. Describe the setting, locations, and relevant dates, including periods of recruitment, exposure, follow-up, and data collection, when available.	Section 2.2 and 2.9	GWAS summary statistics for hypothalamic volume and its five subregions were obtained from a previous MRI-based GWAS involving 32,956 individuals. The aortic aneurysm outcome data were obtained from the FinnGen R9 release (finn-b-I9_AORTANEUR), which integrates genomic data with nationwide health registry information from the Finnish population. Gene expression regulatory data were obtained from the eQTLGen consortium, which provides whole-blood cis-eQTL summary statistics from 31,684 individuals of predominantly European ancestry.

		b)	Participants: Give the eligibility criteria, and the sources and methods of selection of participants. Report the sample size, and whether any power or sample size calculations were carried out prior to the main analysis	Section 2.2 and 2.9	The present study did not involve direct recruitment of participants for the genetic analyses. Instead, publicly available summary-level GWAS and cis-eQTL datasets were used. The hypothalamic volume GWAS included 32,956 participants with available MRI and genetic data. The FinnGen aortic aneurysm dataset included 2,825 cases and 206,541 controls, with cases defined based on ICD-10 code I71. The eQTLGen dataset comprised 31,684 whole-blood samples from individuals of European ancestry. No additional sample size calculation was performed because this study was based on publicly available summary statistics.
		c)	Describe measurement, quality control and selection of genetic variants	Section 1	In order to obtain reliable findings, this study was based on three major assumptions underlying MR studies. First, the instrumental variables must exhibit a strong correlation with the exposure. Second, the instrumental variables should not be associated with the outcome. Third, the instrumental variables must be independent of confounding factors.
		d)	For each exposure, outcome, and other relevant variables, describe methods of assessment and diagnostic criteria for diseases	Section 2.2	The classification of aortic aneurysm cases was based on the ICD-10 code I71 from the National Hospitalization and Death Registry and related records, while the control group consisted of individuals who did not meet this phenotypic criterion.
		e)	Provide details of ethics committee approval and participant informed consent, if relevant	NA	NA
5	**Assumptions**		Explicitly state the three core IV assumptions for the main analysis (relevance, independence and exclusion restriction) as well assumptions for any additional or sensitivity analysis	Section 1	In order to obtain reliable findings, this study was based on three major assumptions underlying MR studies. First, the instrumental variables must exhibit a strong correlation with the exposure. Second, the instrumental variables should not be associated with the outcome.

					Third, the instrumental variables must be independent of confounding factors.
6	**Statistical methods: main analysis**		Describe statistical methods and statistics used		
		a)	Describe how quantitative variables were handled in the analyses (i.e., scale, units, model)		The quantitative exposure variables encompassed the total volume of the hypothalamus and the volumes of its five subregions, which were examined as continuous traits utilizing GWAS summary statistics. The effects of single nucleotide polymorphisms (SNPs) were quantified per standard deviation (SD) increase in regional brain volume. In the mediation Mendelian randomization analyses, gene expression levels obtained from the eQTLGen consortium were considered as quantitative mediators, with eQTL effect sizes denoting changes per allele in normalized gene expression. The outcome variable, aortic aneurysm, was assessed as a binary trait on the log-odds scale, with results presented as odds ratios (ORs) accompanied by 95% confidence intervals.
		b)	Describe how genetic variants were handled in the analyses and, if applicable, how their weights were selected	Section 2.3 and 2.9	To ensure the robustness and sensitivity of the analytical outcomes, significance thresholds of $P < 5 \times 10^{-8}$ or $P < 5 \times 10^{-6}$ were employed across various analyses. Only SNPs with a minor allele frequency (MAF) greater than 0.01 were considered.
		c)	Describe the MR estimator (e.g. two-stage least squares, Wald ratio) and related statistics. Detail the included covariates and, in case of two-sample MR, whether the same covariate set was used for adjustment in the two samples	Section 2.3 and 2.9	We excluded SNPs with $R2<0.001$ using linkage disequilibrium (LD) analysis. To avoid weak instrument bias. An F-statistic value >10 was regarded as strong enough to avoid weak instrument bias. A significance level of FDR-corrected $p < 0.05$ was considered significant.

		d)	Explain how missing data were addressed	Section 2.3	For both exposure and outcome GWAS datasets, SNPs with missing information (e.g., effect sizes, standard errors, or allele details) were excluded prior to harmonization. No imputation of missing data was performed. Sensitivity analyses were conducted to ensure that exclusion of SNPs with incomplete data did not materially affect the results.
		e)	If applicable, indicate how multiple testing was addressed	Section 2.3 and 2.11	The false discovery rate (FDR) based on the Benjamini-Hochberg approach was used for multiple testing corrections. A significance level of FDR-corrected $p < 0.05$ was considered significant.
7	**Assessment of assumptions**		Describe any methods or prior knowledge used to assess the assumptions or justify their validity	Section 2.3 and 2.9	An F-statistic value >10 was regarded as strong enough to avoid weak instrument bias. All IVs in this study had an F-statistic >10. The false discovery rate (FDR) based on the Benjamini-Hochberg approach was used for multiple testing corrections. A significance level of FDR-corrected $p < 0.05$ was considered significant.
8	**Sensitivity analyses and additional analyses**		Describe any sensitivity analyses or additional analyses performed (e.g. comparison of effect estimates from different approaches, independent replication, bias analytic techniques, validation of instruments, simulations)	Section 2.3	We conducted sensitivity analyses utilizing the MR-Egger method, along with the weighted median and weighted mode techniques, to ensure the robustness of our findings. The heterogeneity of the instrument variable was determined by Cochran's Q statistic. To assess horizontal pleiotropy, we used the MR-Egger intercept analysis. Furthermore, we performed an MR-PRESSO analysis to identify and correct for the presence of specific aberrant instrumental variables. In addition, Steiger filtering was applied to verify the causal direction, ensuring that the genetic variants explained more variance in the exposure than in the outcome. A significance level of FDR-corrected $p < 0.05$ was considered significant.

9	**Software and pre-registration**				
		a)	Name statistical software and package(s), including version and settings used	Section 2.3	All tests were two-sided and analyzed using the TwoSampleMR packages (https://github.com/MRCIEU/TwoSampleMR) in R software.
		b)	State whether the study protocol and details were pre-registered (as well as when and where)	NA	NA
	**RESULTS**				
10	**Descriptive data**				
		a)	Report the numbers of individuals at each stage of included studies and reasons for exclusion. Consider use of a flow diagram	Section 2.3 and 2.9	This study utilized publicly available summary-level GWAS and cis-eQTL datasets, and no additional participant recruitment, follow-up, or individual-level exclusion procedures were performed. The included datasets comprised hypothalamic subregional volume GWAS data from 32,956 individuals, aortic aneurysm GWAS data from FinnGen R9 including 2,825 cases and 206,541 controls, and eQTLGen cis-eQTL data from 31,684 individuals of predominantly European ancestry. As the analyses were based on previously generated summary statistics, there were no sequential stages of participant inclusion or exclusion within the present study, and a traditional participant flow diagram was not applicable.
		b)	Report summary statistics for phenotypic exposure(s), outcome(s), and other relevant variables (e.g. means, SDs, proportions)	Section 2.3 and 2.9	Summary statistics were derived from large-scale genome-wide association studies (GWASs). GWAS summary statistics for hypothalamic volume and its five subregions were obtained from a previous study that utilized MRI and genetic data from 32,956 individuals of European ancestry, with regional brain volumes standardized (z-scored) prior to analysis. Gene expression data were obtained from the eQTLGen consortium, comprising

				31,684 peripheral blood samples of European ancestry, and normalized expression levels were used as quantitative mediators in the mediation MR analyses. Summary statistics for aortic aneurysm were obtained from the FinnGen project (release R9; GWAS ID: finn-b-I9_AORTANEUR), including 2,825 cases and 206,541 controls. As all datasets were based on published summary-level data, individual-level means and standard deviations were not available.
		c) If the data sources include meta-analyses of previous studies, provide the assessments of heterogeneity across these studies	NA	NA
		d) For two-sample MR:<br>i. Provide justification of the similarity of the genetic variant-exposure associations between the exposure and outcome samples<br>ii. Provide information on the number of individuals who overlap between the exposure and outcome studies	Section 2.3 and 2.9	(i) The exposure (hypothalamic volume and subregional volumes) and outcome (aortic aneurysm) GWAS summary statistics were both derived from large-scale studies of European ancestry individuals (UK Biobank and FinnGen, respectively). This population homogeneity minimizes bias due to population stratification and ensures comparability of genetic variant–exposure associations across samples.<br>(ii) The exposure and outcome datasets were obtained from independent cohorts, and no sample overlap is expected between the UK Biobank–based GWAS and the FinnGen consortium data. Therefore, bias due to sample overlap is unlikely. The eQTLGen dataset, also based on independent European samples, was used as an intermediate layer in the mediation MR analysis and does not share participants with either GWAS dataset.
11	**Main results**			
		a) Report the associations between genetic variant and exposure, and between genetic variant and outcome, preferably on an interpretable scale		The quantitative exposure variables encompassed the total volume of the hypothalamus and the volumes of its five subregions, which were examined as continuous

					traits utilizing GWAS summary statistics. The effects of SNPs were quantified per standard deviation increase in regional brain volume. In the mediation Mendelian randomization analyses, gene expression levels obtained from the eQTLGen consortium were considered as quantitative mediators, with eQTL effect sizes denoting changes per allele in normalized gene expression. The outcome variable, aortic aneurysm, was assessed as a binary trait on the log-odds scale, with results presented as odds ratios accompanied by 95% confidence intervals.
		b)	Report MR estimates of the relationship between exposure and outcome, and the measures of uncertainty from the MR analysis, on an interpretable scale, such as odds ratio or relative risk per SD difference	Table S13	MR estimates for the hypothalamic volume–aortic aneurysm association were reported as odds ratios with 95% CI per 1 SD increase in exposure. For mediation MR analyses, exposure–mediator and mediator–outcome effects, as well as indirect and direct effects, were expressed as β coefficients with standard errors. Statistical significance was set at $P < 0.05$.
		c)	If relevant, consider translating estimates of relative risk into absolute risk for a meaningful time period	NA	NA
		d)	Consider plots to visualize results (e.g. forest plot, scatterplot of associations between genetic variants and outcome versus between genetic variants and exposure)	Fig. S1	Refer to Supplement 2 for detailed results.
12	**Assessment of assumptions**				
		a)	Report the assessment of the validity of the assumptions	Section 3.1, Supplement 1	This paper reports the results of the assessment of the validity of the associated hypotheses in several places throughout the text and in the supplementary information. For example, In Table S3, the heterogeneity of the statistical model was tested with the Q-statistic to assess its stability, and MR-Egger intercept analysis was used to assess horizontal pleiotropy.

		b)	Report any additional statistics (e.g., assessments of heterogeneity across genetic variants, such as I^2, Q statistic or E-value)	Supplement 1	Detailed information can be found in Supplement 1
13	**Sensitivity analyses and additional analyses**				
		a)	Report any sensitivity analyses to assess the robustness of the main results to violations of the assumptions	Section 3.1	Bidirectional MR analysis and sensitivity analysis
		b)	Report results from other sensitivity analyses or additional analyses	NA	NA
		c)	Report any assessment of direction of causal relationship (e.g., bidirectional MR)	Fig. S1	Fig. S1
		d)	When relevant, report and compare with estimates from non-MR analyses	Fig. 2	The findings from the MRI analysis corroborate the results obtained from the MR analysis.
		e)	Consider additional plots to visualize results (e.g., leave-one-out analyses)	NA	NA
	**DISCUSSION**				
14	**Key results**		Summarize key results with reference to study objectives	Section 5	The MR analysis indicates a causal relationship between the reduction in superior tubular volume and an elevated risk of aortic aneurysm. Utilizing MRI data, we further identified a reduction in superior tubular volume in patients with aortic aneurysms, with the most pronounced changes observed in the right superior tubular region. Importantly, our findings reveal that IL1B partially mediates the impact of hypothalamic atrophy on the susceptibility to aortic aneurysms.
15	**Limitations**		Discuss limitations of the study, taking into account the validity of the IV assumptions, other sources of potential bias, and imprecision. Discuss both direction and magnitude of any potential bias and any efforts to address them	Section 4	the Mendelian Randomization analysis relies on publicly accessible databases and genetic instrumental variables. While this approach can mitigate confounding factors to some extent, the effectiveness and specificity of the instrumental

					variables may be limited, and the potential impact of horizontal pleiotropy on the results cannot be entirely ruled out
16	**Interpretation**				
		a)	Meaning: Give a cautious overall interpretation of results in the context of their limitations and in comparison with other studies	Section 4	The entry is discussed at maximum length in the example text, which rationalizes the MR results by comparing them to multiple published studies.
		b)	Mechanism: Discuss underlying biological mechanisms that could drive a potential causal relationship between the investigated exposure and the outcome, and whether the gene-environment equivalence assumption is reasonable. Use causal language carefully, clarifying that IV estimates may provide causal effects only under certain assumptions	Section 4	The discussion section explores the biological mechanisms that might explain the causal link between exposure and outcome.
		c)	Clinical relevance: Discuss whether the results have clinical or public policy relevance, and to what extent they inform effect sizes of possible interventions	Section 5	Our results suggest that structural alterations in the hypothalamus may play a causal role in the onset and progression of aortic aneurysms via inflammatory pathways, such as IL1B. This provides novel insights into the pathophysiological mechanisms underlying aortic aneurysms and highlights potential therapeutic interventions targeting inflammation.
17	**Generalizability**		Discuss the generalizability of the study results (a) to other populations, (b) across other exposure periods/timings, and (c) across other levels of exposure		our data source is mainly European population, which makes our findings not directly applicable to others
	**OTHER INFORMATION**				
18	**Funding**		Describe sources of funding and the role of funders in the present study and, if applicable, sources of funding for the databases and original study or studies on which the present study is based	Section 9	This study was supported by the Beijing New Health Industry Development Foundation (XM2020-02-002) to BY. BY oversaw the research and data analysis.
19	**Data and data sharing**		Provide the data used to perform all analyses or report where and how the data can be accessed, and reference these sources in the article. Provide	Section 2.3 and 2.9	Data sources are publicly available and described in the Materials and Methods section, including UK Biobank, FinnGen (R9), and eQTLGen. All sources are cited in the

		the statistical code needed to reproduce the results in the article, or report whether the code is publicly accessible and if so, where		manuscript. Analytical code is available from the corresponding author upon reasonable request.
20	**Conflicts of Interest**	All authors should declare all potential conflicts of interest	Section 8	The authors declare that they have no competing interests.

This checklist is copyrighted by the Equator Network under the Creative Commons Attribution 3.0 Unported (CC BY 3.0) license.

1. Skrivankova VW, Richmond RC, Woolf BAR, Yarmolinsky J, Davies NM, Swanson SA, et al. Strengthening the Reporting of Observational Studies in Epidemiology using Mendelian Randomization (STROBE-MR) Statement. JAMA. 2021;under review.

2. Skrivankova VW, Richmond RC, Woolf BAR, Davies NM, Swanson SA, VanderWeele TJ, et al. Strengthening the Reporting of Observational Studies in Epidemiology using Mendelian Randomisation (STROBE-MR): Explanation and Elaboration. BMJ. 2021;375:n2233.
